# Supplementary material for: Controlling the fluorescence and room-temperature phosphorescence behaviour of carbon nanodots with inorganic crystalline nanocomposites
Source: Nat Commun. 2019 Jan 14;10:206. doi: 10.1038/s41467-018-08214-6 (PMC6331607; doi:10.1038/s41467-018-08214-6)
Supplement: Supplementary file 1 — Supplementary Information [file 41467_2018_8214_MOESM1_ESM.pdf]

Supplementary Information

Controlling the fluorescence and room-temperature phosphorescence behaviour of carbon nanodots with inorganic crystalline nanocomposites

David C. Green[1]*, Mark A. Holden[1,2], Mark A. Levenstein[1,3], Shuheng Zhang[1], Benjamin R. G. Johnson[2], Julia Gala de Pablo[2], Andrew Ward[4], Stanley W. Botchway[4], and Fiona C. Meldrum[1]*

[1]School of Chemistry, University of Leeds, LS2 9JT, UK

[2]School of Physics, University of Leeds, LS2 9JT, UK

[3]School of Mechanical Engineering, University of Leeds, LS2 9JT, UK

[4]Central Laser Facility, Science and Technology Facilities Council, Research Complex at Harwell, Rutherford Appleton Laboratory, Didcot OX11 0QX, UK

Supplementary Notes

Supplementary Note 1: *Analysis of XPS data*

XPS provided elemental analysis of the CND samples, which are given below in Supplementary Table 5. There were similar amounts of carbon and oxygen in each sample, whereas F-CNDs contained a higher amount of nitrogen. Both samples contained an amount of sodium, which was expected since NaOH was added in order to form a precursor solution prior to heating. Both samples contained trace chlorine, possibly as impurities in the initial precursor materials.

For each element (N, C and O), a range of local environments and bonding could be determined. Peaks were modelled with the minimum number of Gaussian fits where a good agreement between modelled and real data was obtained.

For F-CNDs, N 1*s* yielded a single, symmetrical, broad peak at 399.7 cm^{-1}, which was modelled with a single fit. The energy of this peak matches to that of N-heterocycles, including pyrrolic and pyridinic N, and is likely to have a contribution from both, although it is not possible here to distinguish between them. C 1*s* was fit with 3 distinct peaks, with a predominant C-C peak (285 cm^{-1}), and two smaller peaks (286.2 cm^{-1} and 288.2 cm^{-1}) assigned to C-N and C=N/C=O respectively. O 1*s* yielded a predominant fit at 531.6 cm^{-1}, corresponding to C=O, followed by two progressively higher energy fits at 533.2 cm^{-1} and 535.6 cm^{-1}, assigned to C-OH and O=C-OH respectively. C 1*s* was decidedly more complex, with a richer variety of bonding for carbon compared to F-CNDs.

For R-CNDs, N 1*s* yielded two distinct peaks at 399.4 cm^{-1} and 400.9 cm^{-1}, which were assigned to N-heterocycles and C=N respectively. The latter peak was more intense, suggesting a much smaller proportion of the heterocycles compared to F-CNDs.

Importantly, XPS revealed no graphitic carbon, therefore the product materials cannot be true carbon quantum dots. This absence of graphite was confirmed by TEM (Supplementary Figs. 1 and 15). Although very low density nanoparticles were detected, ranging from 3-5 nm in size, no layered graphite structure was seen in their interior. Because of this, the product materials are carbon-rich, amorphous nanoparticles, possibly ensembles of fluorophores around a carbonised core. To signify this, and separate them from true carbon dots, they are referred to as carbon nanodots, or CNDs.[1]

Supplementary Figures

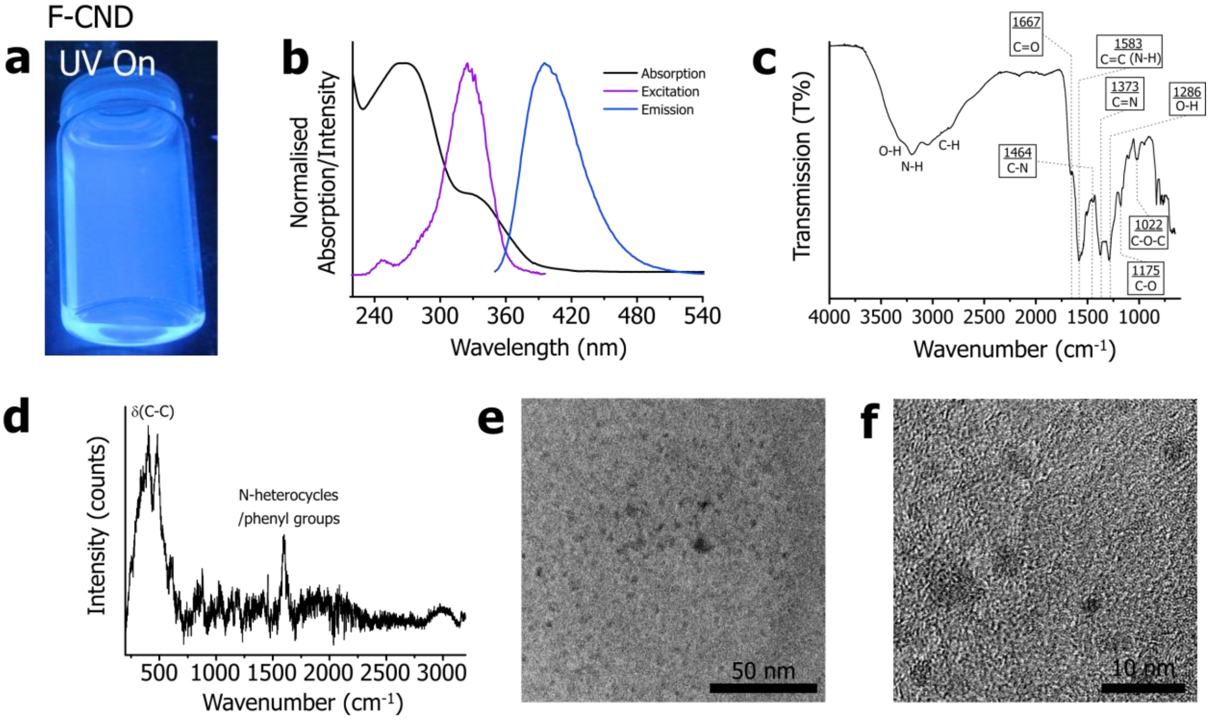

Supplementary Fig. 1: Characterisation of F-CND by (**a**) photography, (**b**) absorbance, excitation (λ_{em} = 400 nm) and emission (λ_{ex} = 320 nm) spectroscopy, (**c**) FTIR spectroscopy, (**d**) Raman spectroscopy and (e and f) TEM. F-CND, as an aqueous solution, fluoresces blue under UV (365 nm) excitation. The lowest energy absorbance peak coincides with the fluorescence excitation maximum at 320 nm, which yields a broad, asymmetric emission maximum at 400 nm (**b**). FTIR (**c**) confirmed the presence of a wide range of functional groups, while Raman (**d**) confirmed a predominance of C-C bonds (as expected for amorphous carbon) and N-heterocycles and phenyl groups associated with folic acid, indicating that these principle moieties are retained. TEM (**e**) revealed a particular size range of 3-5 nm, and high-magnification TEM (**f**) reveals the amorphous carbonaceous core of F-CND (as opposed to graphitic).

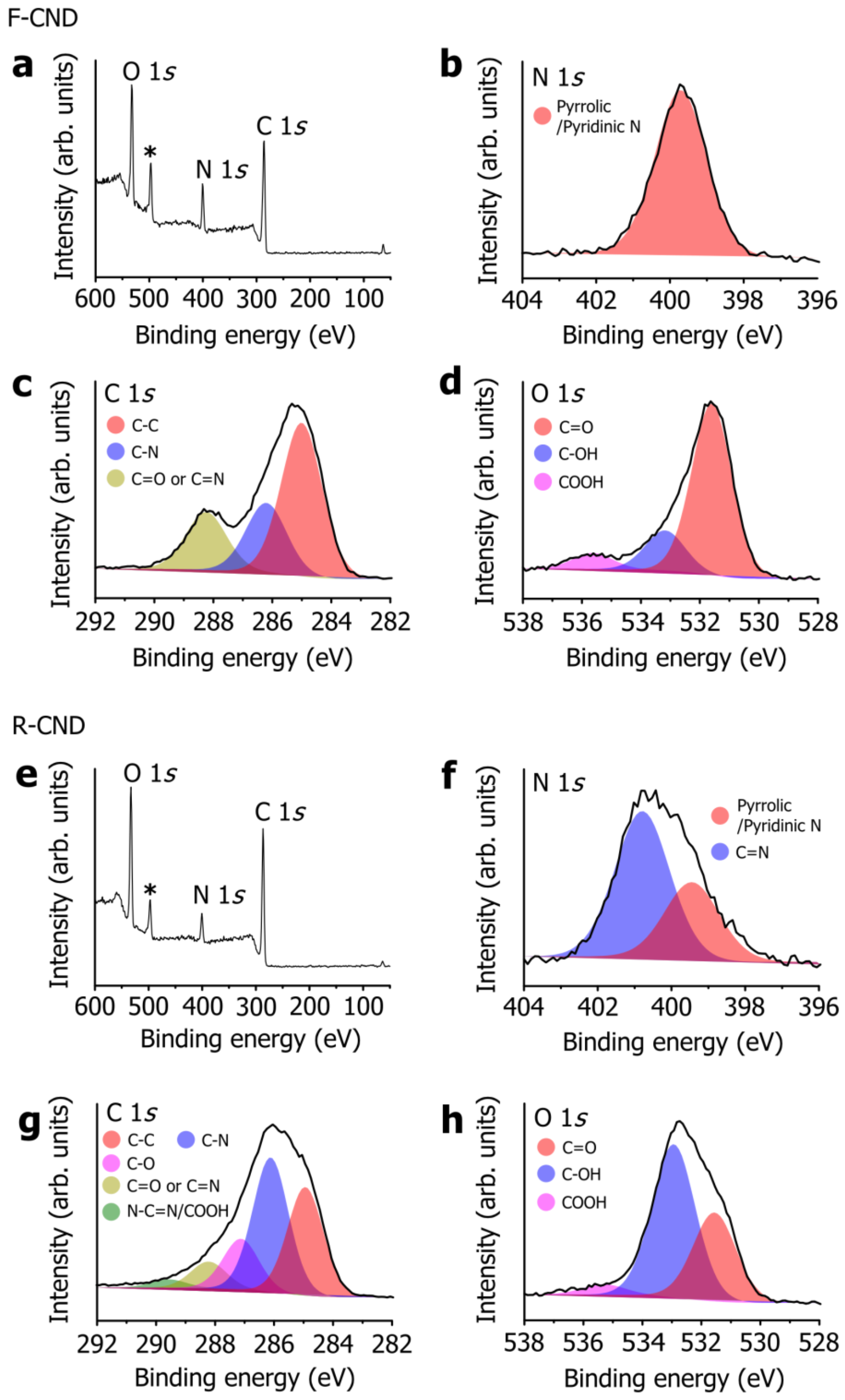

Supplementary Fig. 2: XPS spectra for F-CND (**a-d**) and R-CND (**e-h**). Of the full spectra obtained (**a** and **e**), raw data (solid black lines) for N 1*s* (**b** and **f**), C 1*s* (c and g) and O 1*s* (**d** and **h**) were deconvoluted separately into fitted peaks (coloured area plots). Each corresponding assignment to each fit is presented on each plot in a legend, where different colours represent different local bonding. On full spectra, the Na auger peak is denoted with an asterisk (*).

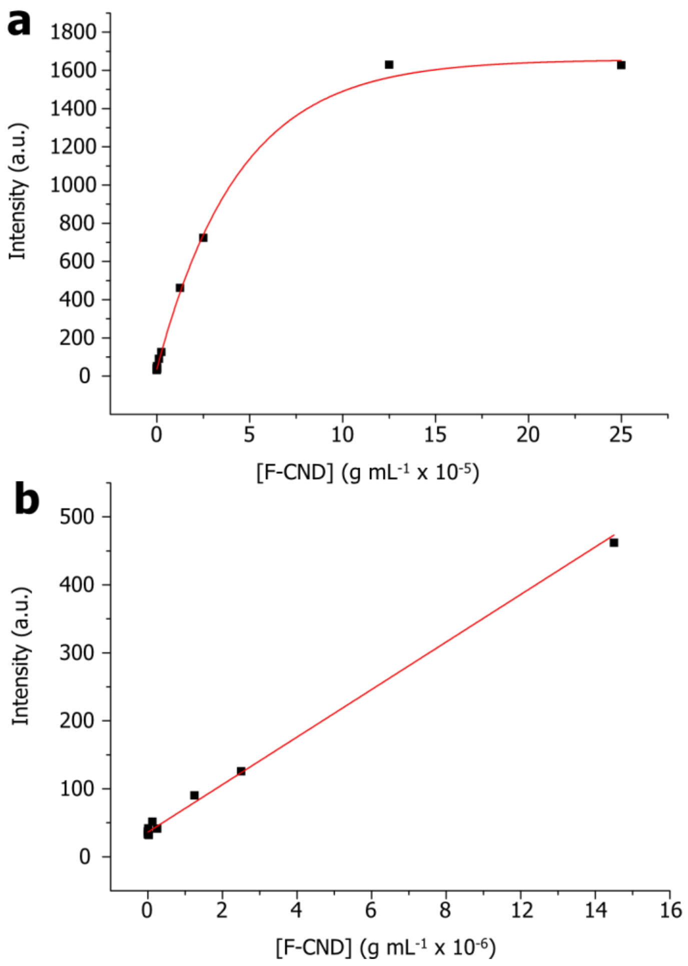

Supplementary Fig.3. Full range of calibration curve (**a**, fluorescence intensity vs. concentration (g mL^{-1}) of F-CND aqueous solutions, revealing a self-quenching threshold. The linear relationship between fluorescence intensity and F-CND concentration is marked with a blue circle in **a**, and expanded in **b**.

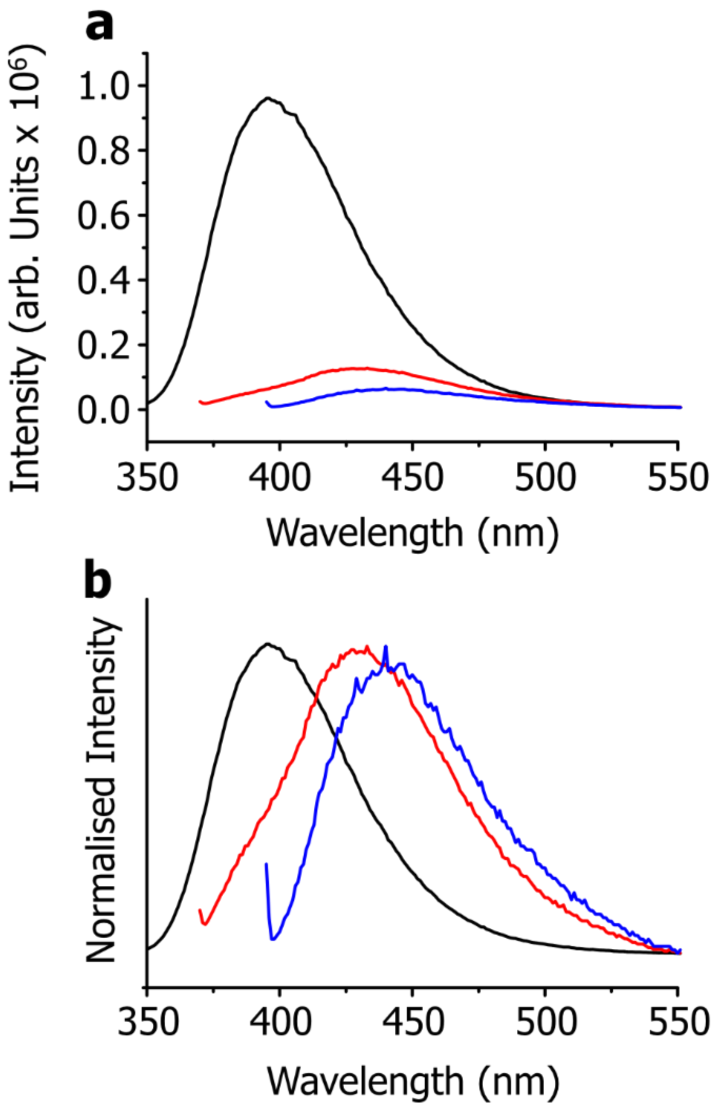

Supplementary Fig. 4. Emission spectra obtained from aqueous F-CND solutions (**a**, λ_{ex} = 320 (black), 365 (red) and 390 (blue)) showing a progressive drop in intensity and red shift. Normalised intensity plots highlight the red shift observed with red-shifted excitation wavelengths (**b**, λ_{ex} = 320 (black), 365 (red) and 390 (blue)).

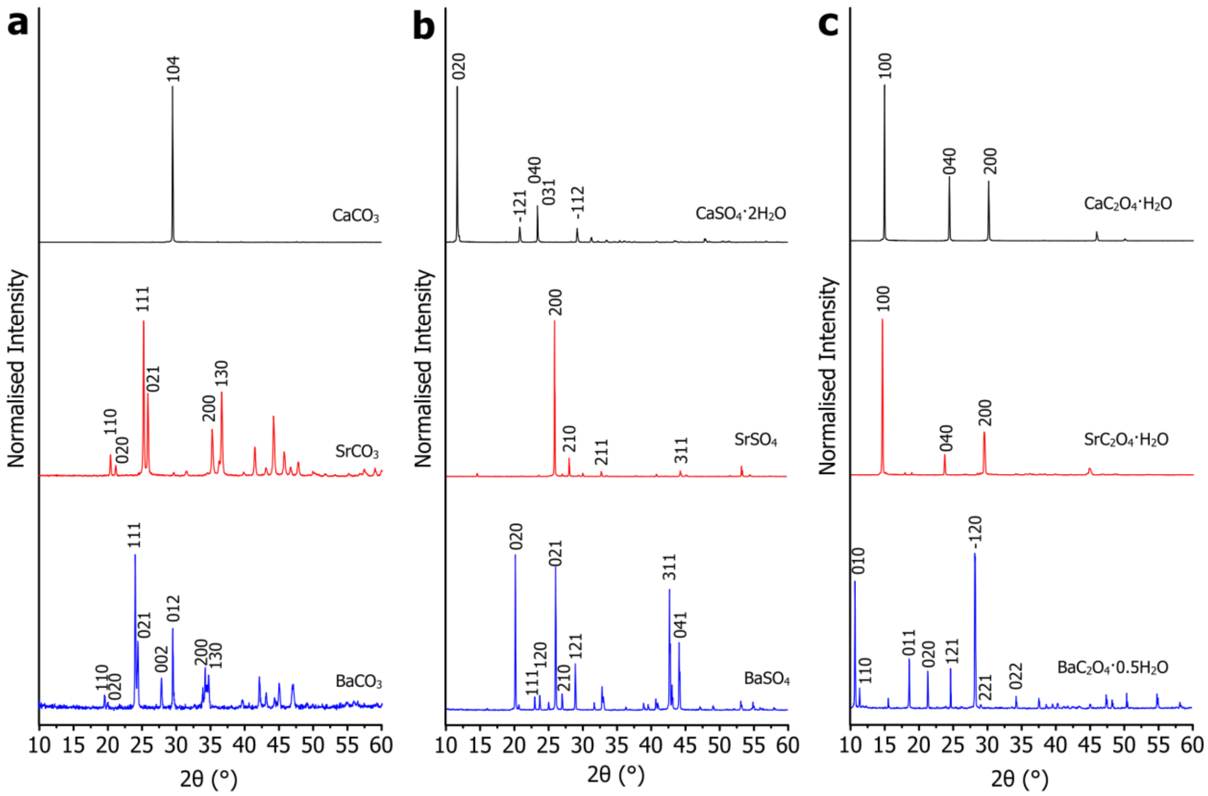

Supplementary Fig. 5. Indexed powder X-ray diffraction (pXRD) patterns for carbonates (**a**), sulfates (**b**) and oxalates (**c**) of Ca (black), Sr (red), and Ba (blue).

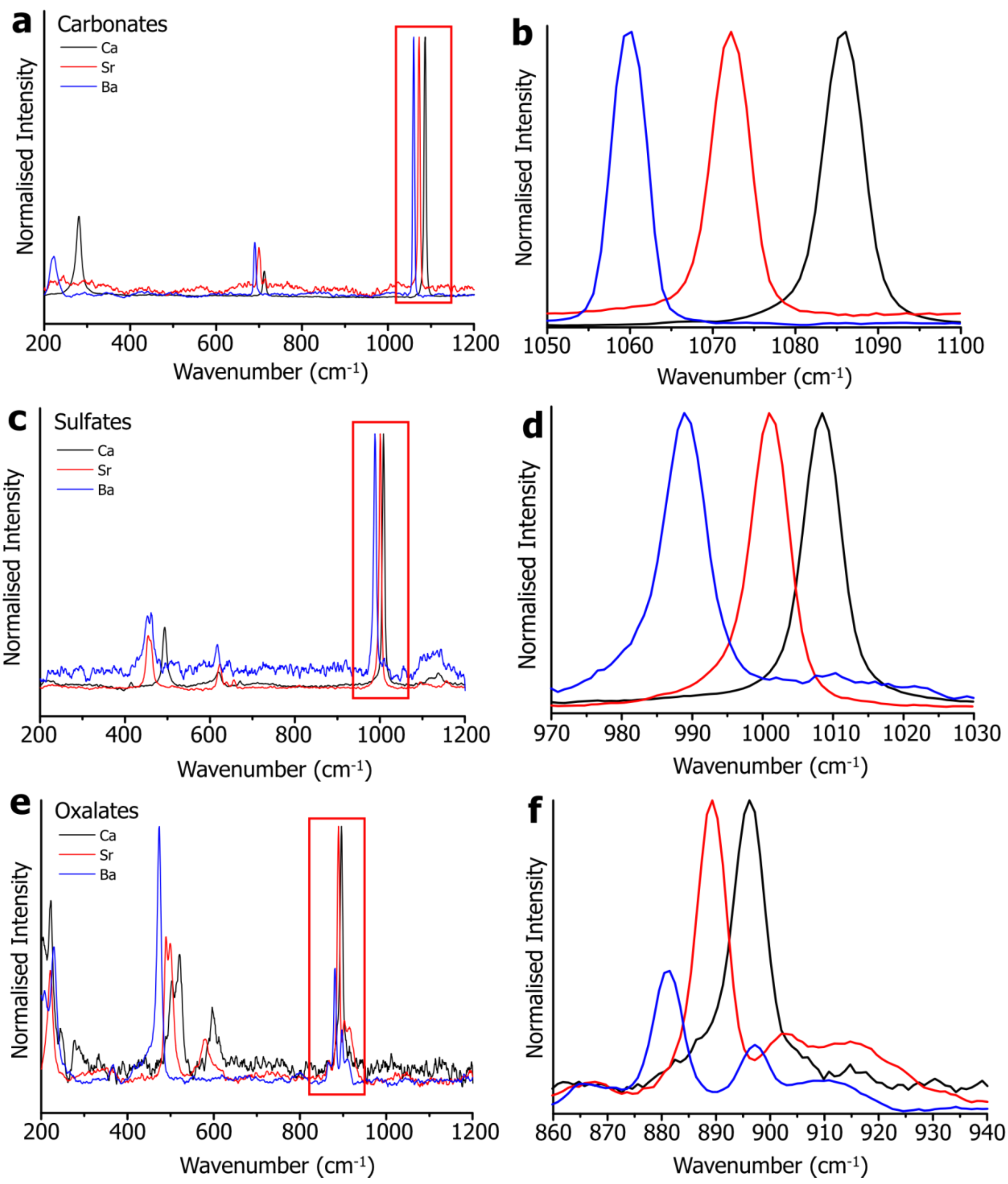

Supplementary Fig. 6. Raman spectra of F-CND/carbonate (**a** and **b**), sulfate (**c** and **d**) and oxalate (**e** and **f**) nanocomposites. The spectra in **a, c** and **e** were used to identify the crystalline product phase alongside pXRD data. Principal active Raman modes as highlighted with a red box in **a, c** and **e** were red shifted with increasing atomic mass of the cation (Ca = Black, Sr = Red and Ba = Blue) (**b**, **d** and **f**).

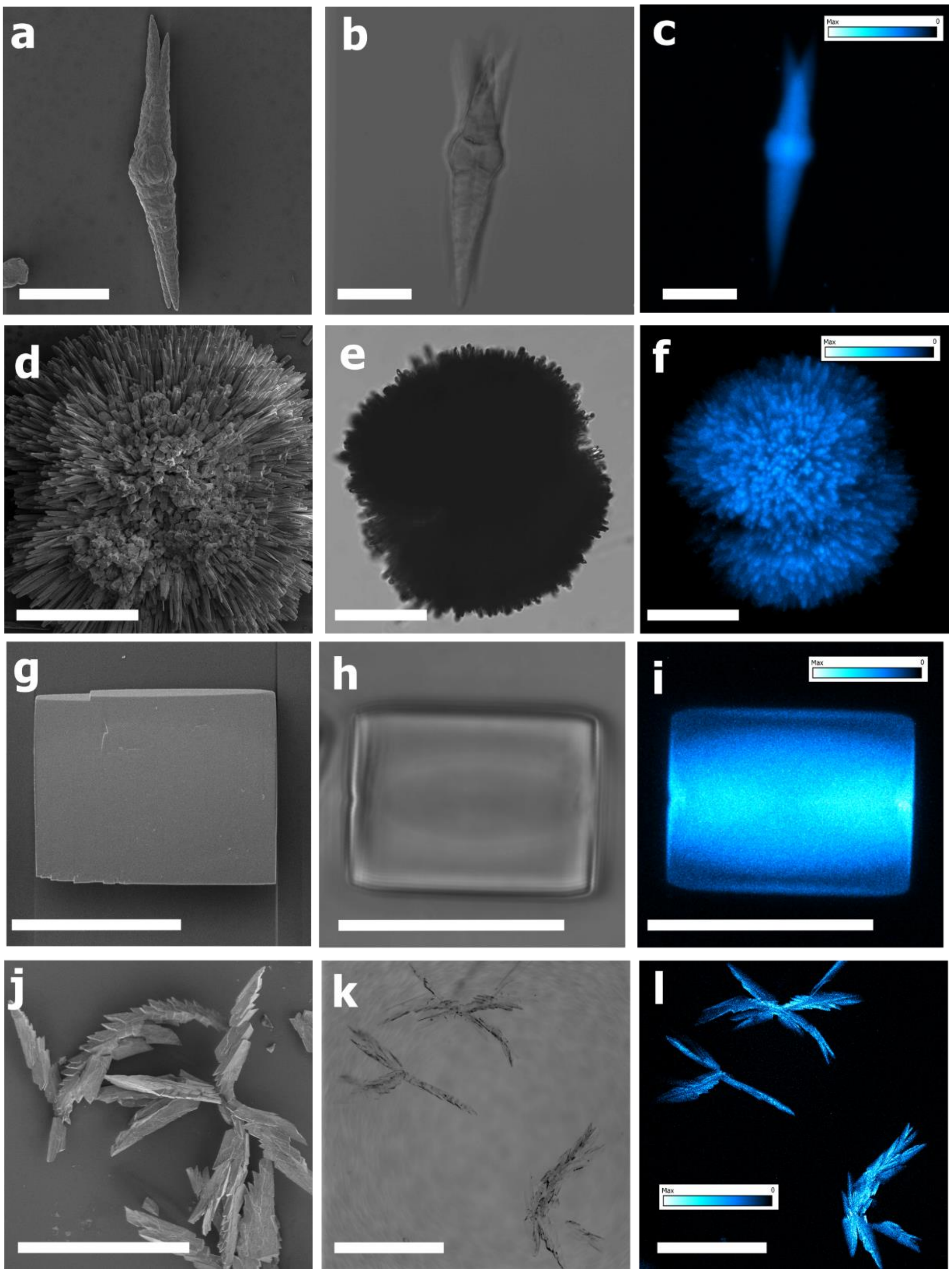

Supplementary Fig. 7. Scanning electron microscopy (SEM) (**a, d, g**, and **j**), optical (**b, e, h** and **k**) and confocal fluorescence microscopy (CFM)(**c, f, i** and **l**) images of $SrCO_3$ (**a-c**), $BaCO_3$ (**d-f**), $BaSO_4$ (**g-i**), and $BaC_2O_4{\cdot}0.5H_2O$ (**j-l**). All CFM images have accompanying look-up table (LUT) scales signifying PL intensity, from white (max) to cyan to blue to black (zero). Scale bars: 10 µm (**a-c, g-i**), 50 µm (**d-f**) and 300 µm (**j-l**).

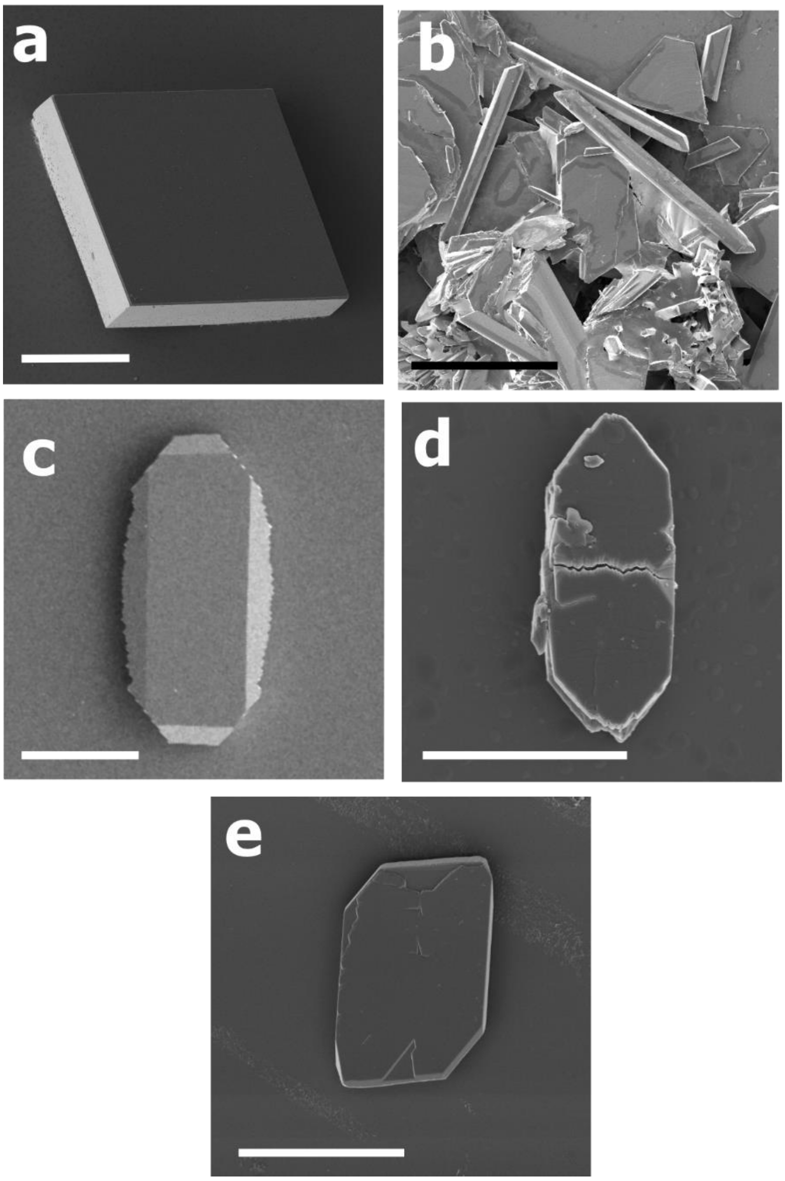

Supplementary Fig. 8. Scanning electron microscopy (SEM) images of $CaCO_3$ (**a**), $CaSO_4{\cdot}2H_2O$ (**b**), $SrSO_4$ (**c**), $CaC_2O_4{\cdot}H_2O$ (**d**), and $SrC_2O_4{\cdot}H_2O$ (**e**). Scale bars: 20 µm (**a** and **c**), 150 µm (**b**), 5 µm (**d**) and 10 µm (**e**).

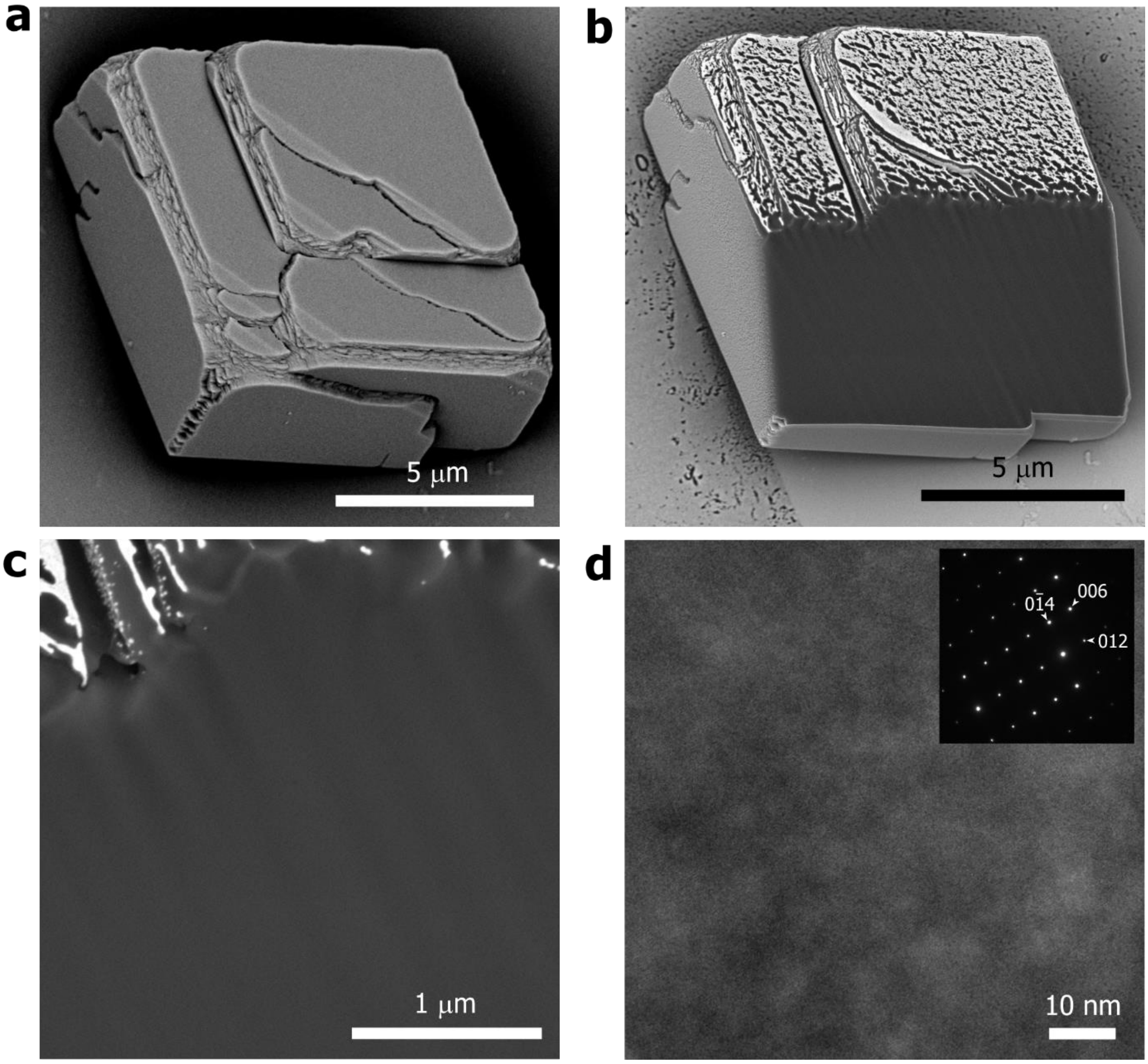

Supplementary Fig. 9: Scanning electron micrographs (**a-c**) and transmission electron micrograph (**d**) of F-CND/$CaCO_3$ nanocomposite containing 0.0376 wt% F-CND. Micrographs show calcite single crystals before milling (**a**) and after Pt-coating and FIB milling (**b**). No trace of incorporated F-CND was detected by SEM (**c**). F-CND embedded in calcite was not detected by TEM (**d**), where sections though a single crystal (as indicated by electron diffraction, see inset) were imaged with no discernible trace of F-CND. Any disruption of the surface is due to beam damage. It is not possible to image the F-CND particles embedded in calcite due to their small sizes, amorphous character, and the greater density and crystallinity of the host crystal.

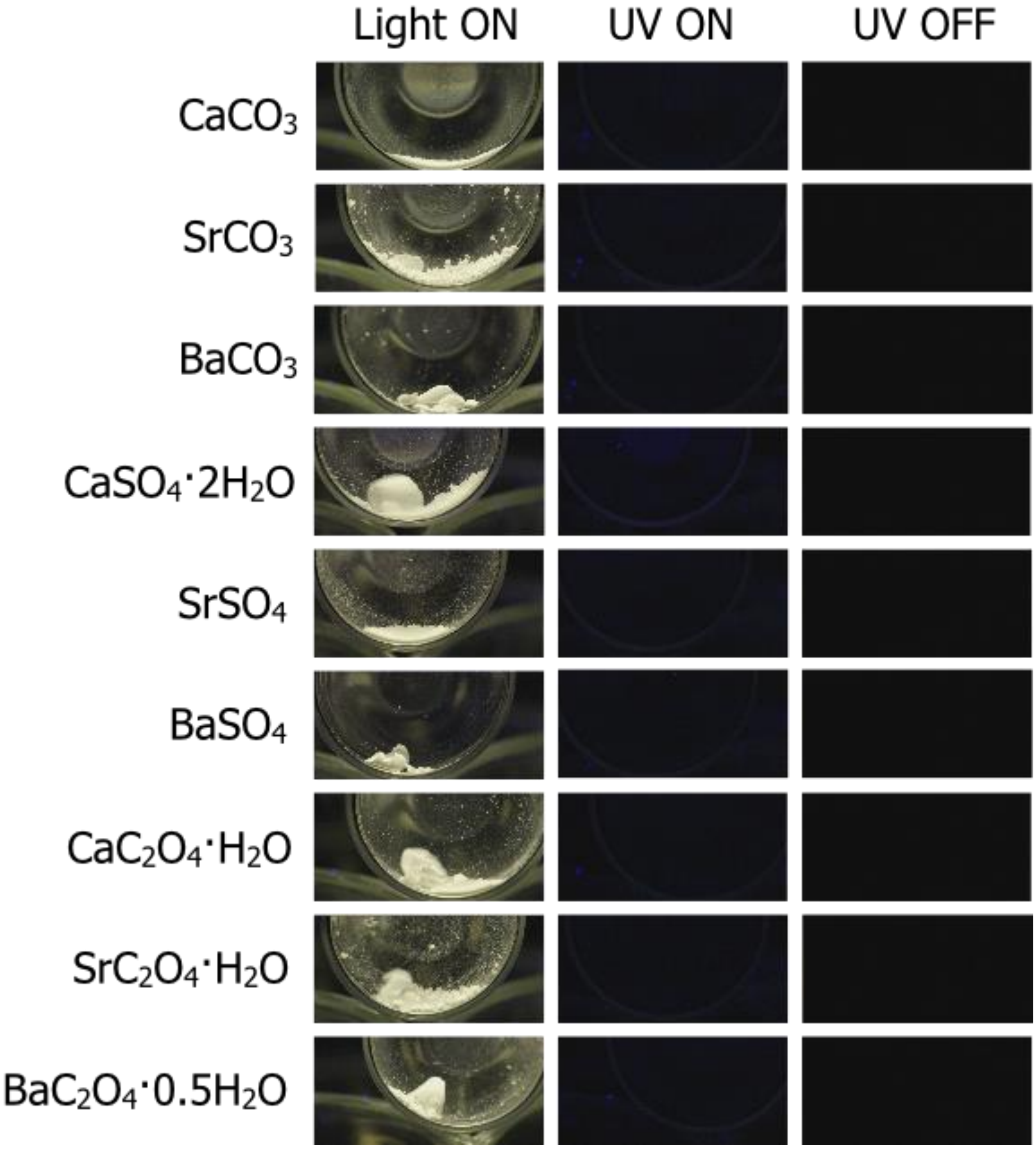

Supplementary Fig. 10: Photographs of inorganic crystalline host phases under normal light, under UV light, and immediately after UV light is switched off. All powders were colourless, and exhibited no intrinsic photoluminescence (fluorescence or phosphorescence).

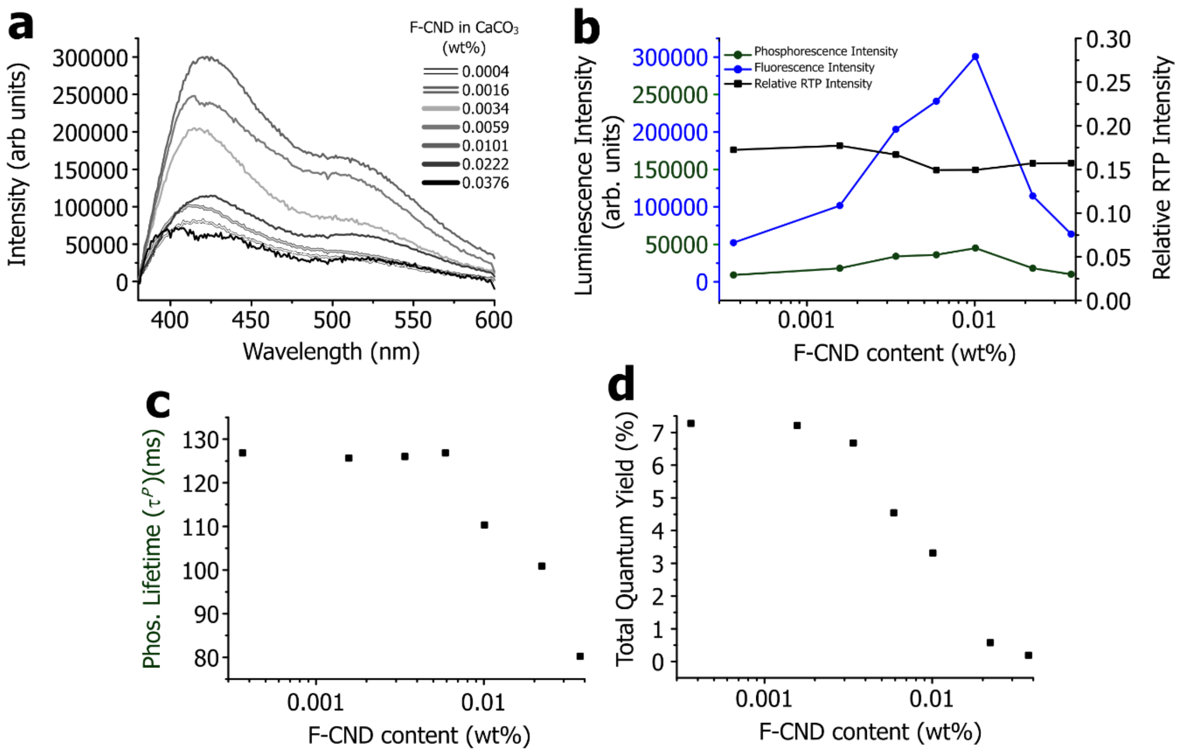

Supplementary Fig. 11: F-CND/$CaCO_3$ self-quenching study examining the influence of increasing content of F-CND in nanocomposites on SS-PL spectra (**a**), relative phosphorescence intensity (**b**), phosphorescence lifetimes (**c**) and the total quantum yield of the photoluminescence (**d**). Background-removed SS-PL spectra show that PL increases with F-CND content before dropping at high concentrations (**a** and **b**), even though the relative RTP intensity remains constant (**b**). Phosphorescence lifetimes obtained from video stroboscopy (**c**) and total PL quantum yields (**d**) remain constant until higher concentrations, where both attributes became smaller. This data suggests that self-quenching enhances radiationless internal conversion leading to destabilisation of both excited singlet and triplet electronic states, while intersystem crossing remains unaffected.

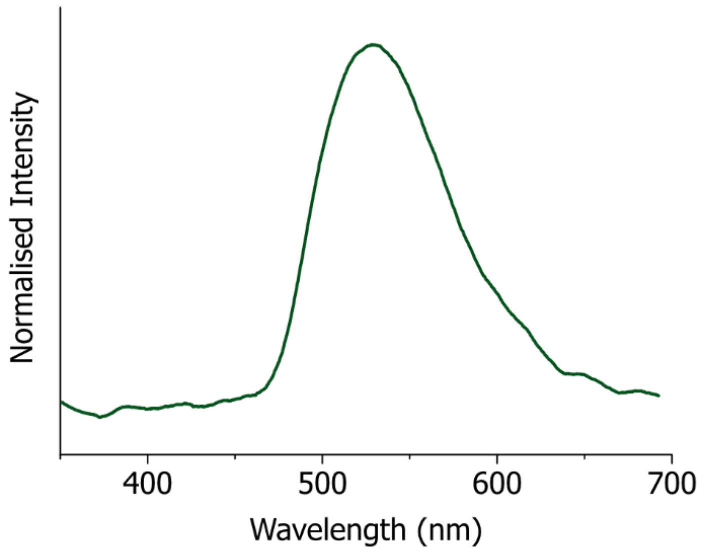

Supplementary Fig. 12. Phosphorescence spectrum (λ_{ex} = 360 nm) of F-CND/$SrSO_4$ nanocomposite.

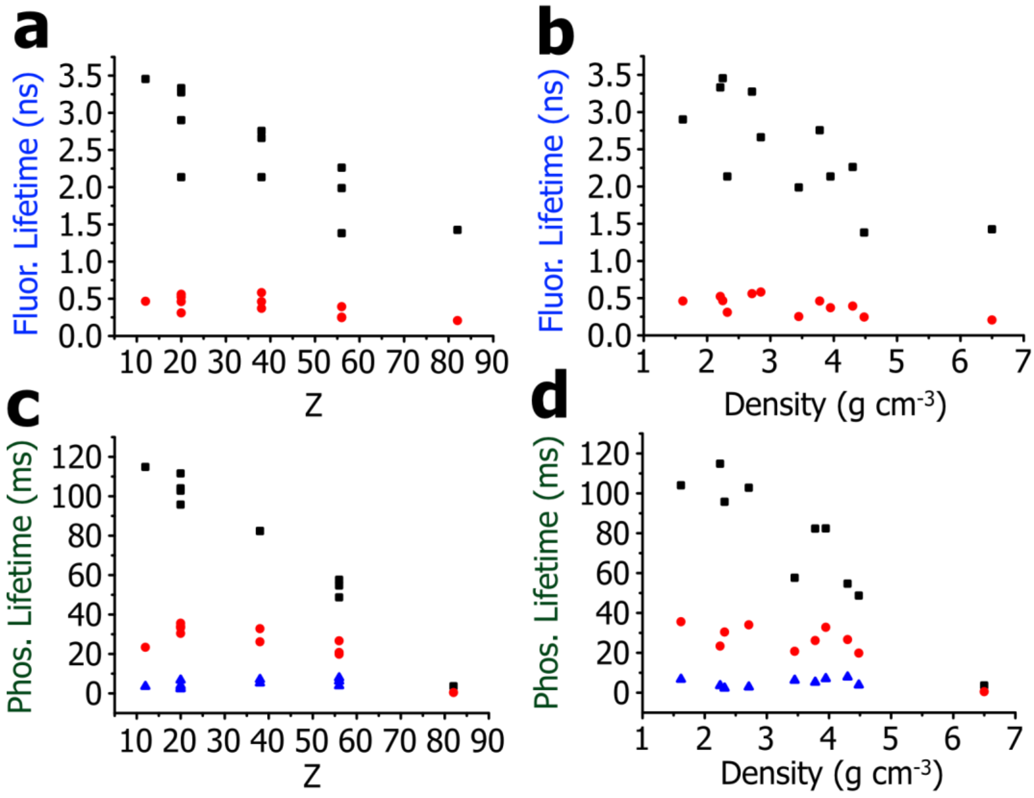

Supplementary Fig. 13. Plots of fluorescence lifetimes fitted from FLIM decay curves (**a** and **b**) and phosphorescence lifetimes fitted from time-resolved phosphorescence microscopy decay curves (**c** and **d**) plotted against host cation Z (**a** and **c**) and density (**b** and **d**). In **a** and **b**, τ_1^F = black square and τ_2^F = red circle. In **c** and **d**, τ_1^P = black square, τ_2^P = red circle and τ_3^P= blue triangle.

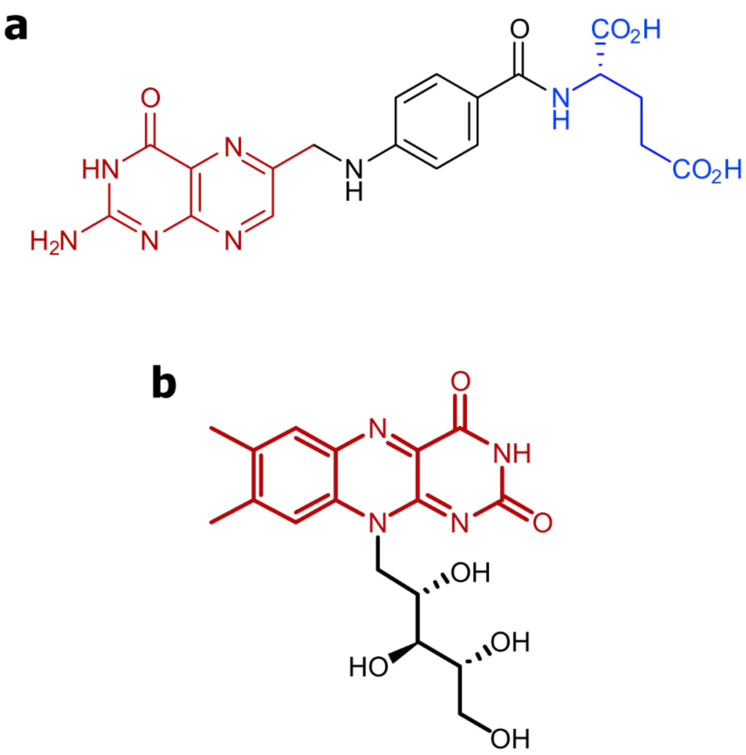

Supplementary Fig. 14. Molecular structures of folic acid (**a**), with the pterin (red) and glutamic acid (blue) moieties coloured for clarity; and riboflavin (**b**) with the flavin (red) moiety coloured for clarity.

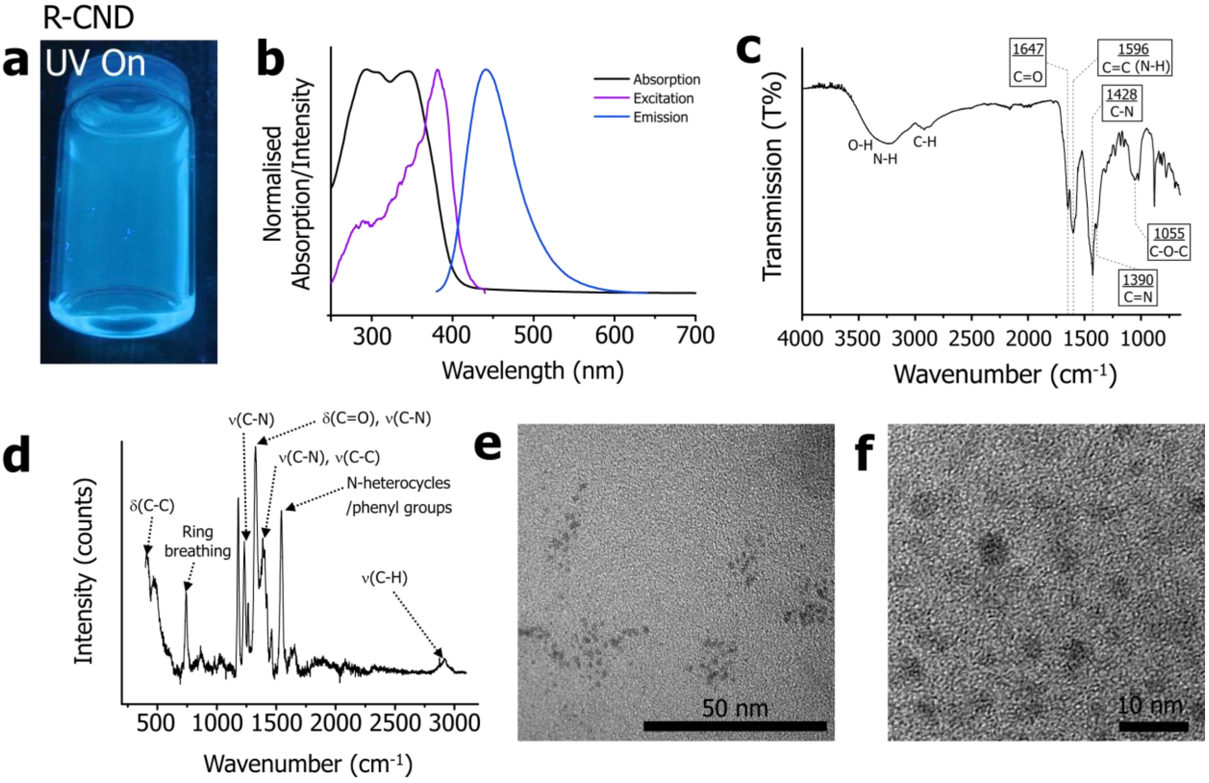

Supplementary Fig. 15: Characterisation of R-CND by photography (**a**); absorbance, excitation (λ_{em} = 440 nm) and emission (λ_{ex} = 380 nm) spectroscopy (**b**), FTIR spectroscopy (**c**), Raman spectroscopy (**d**) and TEM (**e** and **f**). F-CND, as an aqueous solution, fluoresces blue under UV (365 nm) excitation. The lowest energy absorbance coincides with the fluorescence excitation maximum at 380 nm, which yields a broad, asymmetric emission maximum at 440 nm (**b**). FTIR (**c**) confirmed the presence of a wide range of functional groups, but Raman (**d**) confirmed an abundance of C-C bonds and N-heterocycles and phenyl groups associated with riboflavin. TEM (**e**) revealed a particular size range of 3-5 nm, and high-magnification TEM (**f**) revealed the amorphous carbonaceous core of R-CND.

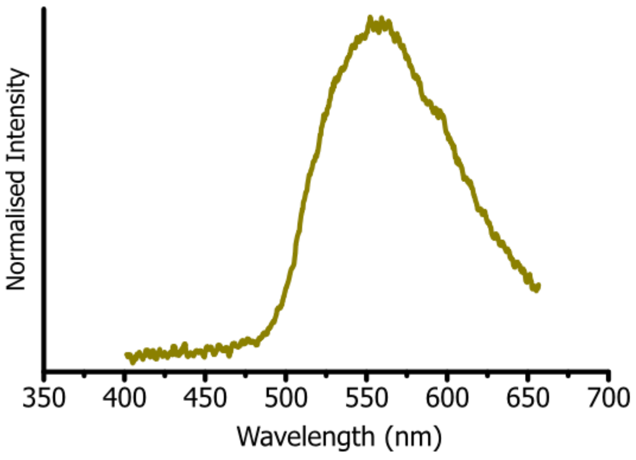

Supplementary Fig. 16: Phosphorescence spectrum (λ_{ex} = 360 nm) of R-CND/$SrSO_4$ nanocomposites.

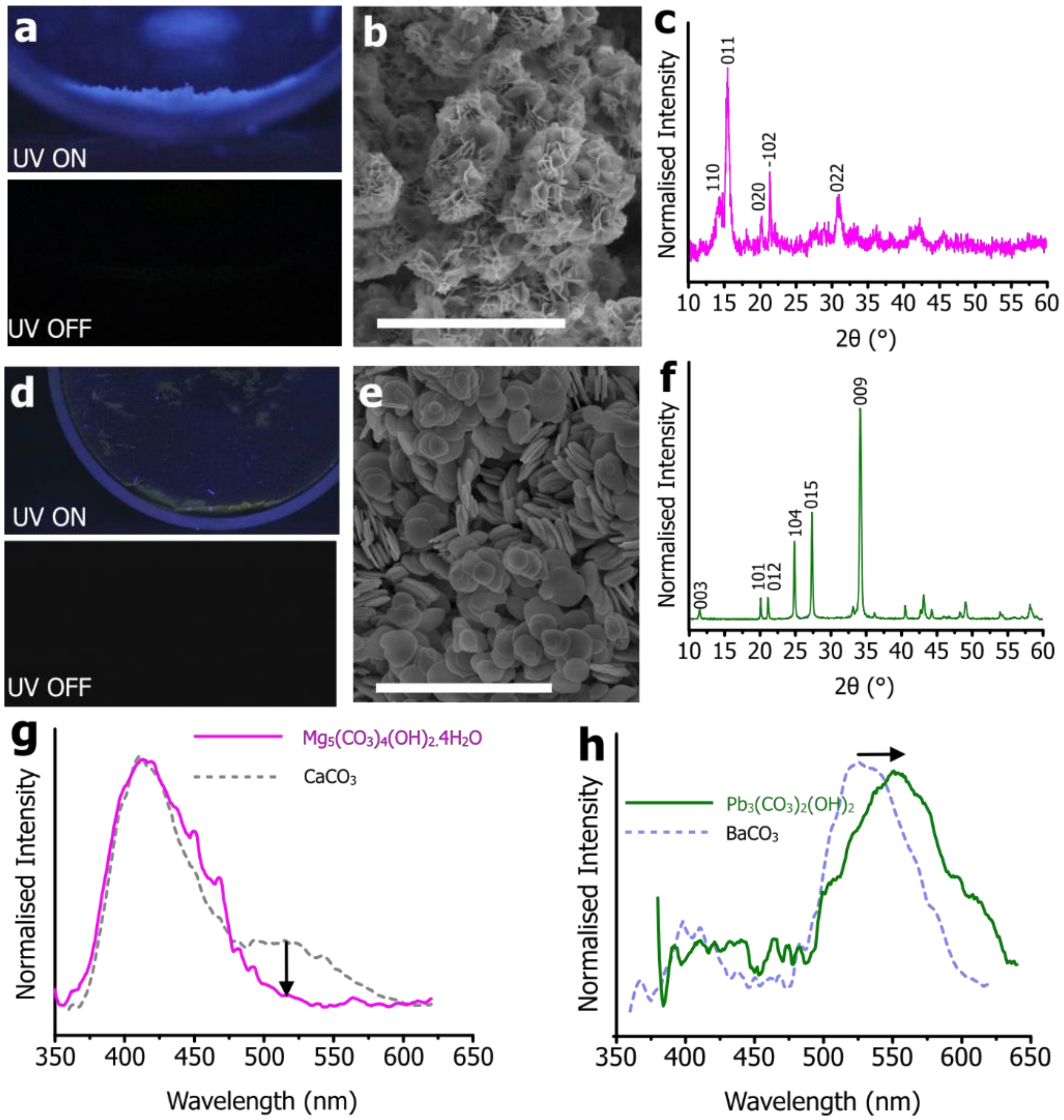

Supplementary Fig. 17. Photographs under UV (365 nm) excitation (UV ON) and immediately after switching off (UV OFF), of F-CND nanocomposites with $Mg_5(CO_3)_4(OH)_2·4H_2O$ (**a**) and $Pb_3(CO_3)_2(OH)_2$ (**d**). SEM micrographs of $Mg_5(CO_3)_4(OH)_2·4H_2O$ (**b**) and $Pb_3(CO_3)_2(OH)_2$ (**e**), and indexed pXRD patterns of $Mg_5(CO_3)_4(OH)_2·4H_2O$ (**c**) and $Pb_3(CO_3)_2(OH)_2$ (**f**). SS-PL emission spectra (λ_{ex} = 320 nm) for $Mg_5(CO_3)_4(OH)_2·4H_2O$ (pink, **g**) and $Pb_3(CO_3)_2(OH)_2$ (green, **h**). The drop in intensity from $CaCO_3$ (shown as black dotted line) to $Mg_5(CO_3)_4(OH)_2.4H_2O$ is highlighted with an arrow. The bathochromic shift of phosphorescence peak from $BaCO_3$ (shown as blue dotted line) to $Pb_3(CO_3)_2(OH)_2$ is highlighted with an arrow. Scale bars: 50 µm (**b**) and 2 µm (**e**).

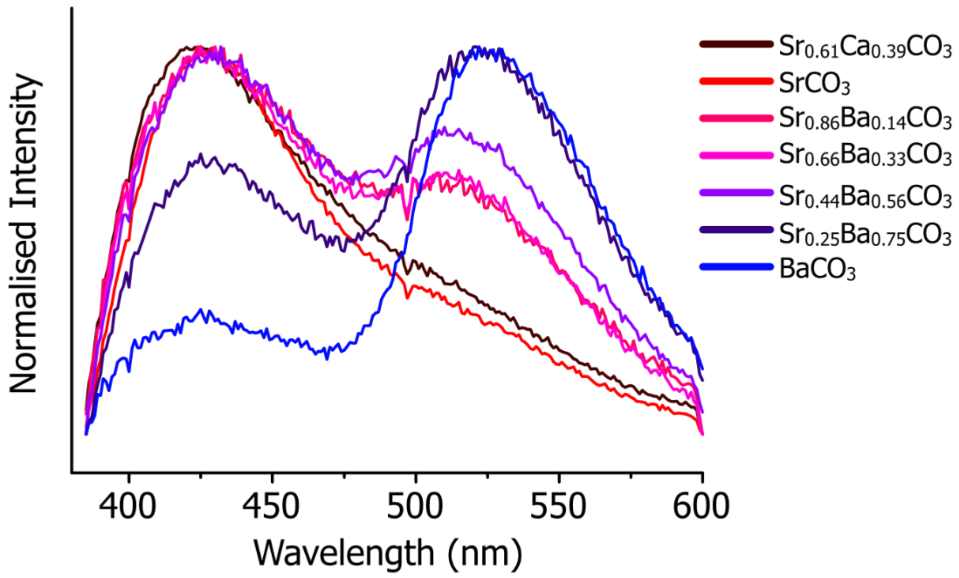

Supplementary Fig. 18: Normalised, background-subtracted SS-PL spectra of F-CND/mixed metal carbonate nanocomposites.

Supplementary Tables

Cation	Anion	Crystalline product identified by pXRD and Raman
Mg^{2+}	CO_3^{2-}	$Mg_5(CO_3)_4(OH)_2 \cdot 4H_2O$ (Hydromagnesite)
Ca^{2+}	CO_3^{2-}	$CaCO_3$ (Calcite)
Sr^{2+}	CO_3^{2-}	$SrCO_3$ (Strontianite)
Ba^{2+}	CO_3^{2-}	$BaCO_3$ (Witherite)
Pb^{2+}	CO_3^{2-}	$Pb_3(CO_3)_2(OH)_2$ (Hydrocerrusite)
Ca^{2+}	SO_4^{2-}	$CaSO_4 \cdot 2H_2O$ (Gypsum)
Sr^{2+}	SO_4^{2}	$SrSO_4$ (Celestine)
Ba^{2+}	SO_4^{2}	$BaSO_4$ (Baryte)
Ca^{2+}	$C_2O_4^{2}$	$CaC_2O_4 \cdot H_2O$ (Whewellite)
Sr^{2+}	$C_2O_4^{2}$	$SrC_2O_4 \cdot H_2O$
Ba^{2+}	$C_2O_4^{2}$	$BaC_2O_4 \cdot 0.5H_2O$

Supplementary Table 1: A summary of the formula and trivial name (where available) of crystalline products identified by pXRD and Raman which were precipitated from aqueous solutions various mixtures of cations and anions.

Study	Identifier*	Protocol**	[$CaCl_2$] (mM)	[$SrCl_2$] (mM)	[$BaCl_2$] (mM)	F-CND (mL)	[Na_2CO_3] (mM)
Quenching	"0.0004 wt%"	$CaCO_3$	25.0	0.0	0.0	0.05	25.0
Quenching	"0.0015 wt%"	$CaCO_3$	25.0	0.0	0.0	0.10	25.0
Quenching	"0.0059 wt%"	$CaCO_3$	25.0	0.0	0.0	0.50	25.0
Quenching	"0.0101 wt%"	$CaCO_3$	25.0	0.0	0.0	1.00	25.0
Quenching	"0.0222 wt%"	$CaCO_3$	25.0	0.0	0.0	2.00	25.0
Quenching	"0.0376 wt%"	$CaCO_3$	25.0	0.0	0.0	5.00	25.0
Solid solution	"$Sr_{0.61}Ca_{0.39}CO_3$"	$SrCO_3$	0.5	1.0	0.0	0.2	1.0
Solid solution	"$Sr_{0.86}Ba_{0.14}CO_3$"	$SrCO_3$	0.0	0.9	0.1	0.2	1.0
Solid solution	"$Sr_{0.66}Ba_{0.34}CO_3$"	$SrCO_3$	0.0	0.7	0.3	0.2	1.0
Solid solution	"$Sr_{0.44}Ba_{0.56}CO_3$"	$SrCO_3$	0.0	0.5	0.5	0.2	1.0
Solid solution	"$Sr_{0.25}Ba_{0.75}CO_3$"	$SrCO_3$	0.0	0.3	0.7	0.2	1.0

Supplementary Table 2: Experimental conditions used in quenching and solid solution studies. *signifies the name used manuscript or figures which corresponds to the associated experimental conditions. **signifies which experimental protocol from Supplementary Table 4 formed the basis for the associated experimental conditions, and was followed for sample preparation.

Salt	Mass (g)	Approx. Concentration (mM)
$MgCl_2 \cdot 6H_2O$	0.813	200
$CaCl_2 \cdot 2H_2O$	0.588	200
$SrCl_2 \cdot 6H_2O$	1.066	200
$BaCl_2$	0.833	200
$Pb(NO_3)_2$	0.132	20
Na_2CO_3	0.424	200
$Na_2C_2O_4$	0.536	200
Na_2SO_4	0.568	200
$NaHCO_3$	0.336	200

Supplementary Table 3: A list of stock solutions of various concentrations formed after the dissolution of the stated mass of each salt in 20 mL DI water.

Mineral	Chemical Formula	Crystallisation Vessel	Stirring Speed (rpm)	T (°C)	t (h)
Hydromagnesite	$Mg_5(CO_3)_4(OH)_2{\cdot}4H_2O$	25 mL vial	600	70	0.5
Calcite	$CaCO_3$	20 mL Petri dish	N/A	RT	48
Polycrystalline $CaCO_3$	$CaCO_3$	25 mL vial	600	RT	0.5
Strontianite	$SrCO_3$	400 mL beaker	N/A	RT	48
Witherite	$BaCO_3$	400 mL beaker	N/A	RT	48
Hydrocerrusite	$Pb_3(CO_3)_2(OH)_2$	400 mL beaker	N/A	RT	48
Gypsum	$CaSO_4{\cdot}2H_2O$	25 mL vial	N/A	RT	48
Celestine	$SrSO_4$	20 mL Petri dish	N/A	RT	48
Baryte	$BaSO_4$	400 mL beaker	N/A	RT	48
Whewellite	$CaC_2O_4{\cdot}H_2O$	400 mL beaker	N/A	RT	48
Strontium oxalate monohydrate	$SrC_2O_4{\cdot}H_2O$	400 mL beaker	N/A	RT	48
Barium oxalate hemihydrate	$BaC_2O_4{\cdot}0.5H_2O$	25 mL vial	N/A	RT	48

Supplementary Table 4: A list of experimental conditions and equipment for the precipitation of host/CD composite phases from aqueous solution.

Mineral	Cation solution	Anion solution	Final [Cation] (mM)	Final [Anion] (mM)	Volume cation stock (mL)	Volume CD stock (mL)	Volume DI water (mL)	Volume anion stock (mL)	Final volume (mL)
Hydromagnesite	$MgCl_2$	$NaHCO_3$	20	40	2	0.2	13.8	4	20
Calcite	$CaCl_2$	Na_2CO_3	25	25	1.25	0.2	7.3	1.25	10
Polycrystalline $CaCO_3$	$CaCl_2$	Na_2CO_3	25	25	2.5	0.4	14.6	2.5	20
Strontianite	$SrCl_2$	Na_2CO_3	1	1	1	0.2	197.8	1	200
Witherite	$BaCl_2$	Na_2CO_3	1	1	1	0.2	197.8	1	200
Hydrocerrusite	$Pb(NO_3)_2$	Na_2CO_3	0.5	0.5	5	0.2	194.3	0.5	200
Gypsum	$CaCl_2$	Na_2SO_4	50	50	5	1	9	5	20
Celestine	$SrCl_2$	Na_2SO_4	5	5	0.25	0.2	9.3	0.25	10
Baryte	$BaCl_2$	Na_2SO_4	0.5	0.5	0.5	0.2	198.8	0.5	200
Whewellite	$CaCl_2$	$Na_2C_2O_4$	0.25	0.25	0.25	0.2	199.3	0.25	200
Strontium oxalate monohydrate	$SrCl_2$	$Na_2C_2O_4$	1	1	1	0.2	197.8	1	200
Barium oxalate hemihydrate	$BaCl_2$	$Na_2C_2O_4$	5	5	0.5	0.2	18.8	0.5	20

Supplementary Table 4 (Continued): A list of experimental conditions and equipment for the precipitation of host/CD composite phases from aqueous solution.

Sample	C 1s (%)	Cl 2p (%)	N 1s (%)	Na 1s (%)	O 1s (%)
F-CND	65.7018	0.115605	11.6364	3.35101	19.1952
R-CND	69.7543	0.266014	6.92511	1.90527	21.1493

Supplementary Table 5: Elemental analysis of CND samples obtained by XPS.

Supplementary References

1. Cayuela, A.; Soriano M. L.; Carrillo-Carrión, C.; and Valcárcel, M.; Semiconductor and carbon-based fluorescent nanodots: the need for consistency. *Chem Commun*. **52**, 1311-1326 (2016)
